# Diisobutyl­ammonium tri­phenyl(2-thiolato­acetato-κ^2^*O*,*S*)stannate(IV)

**DOI:** 10.1107/S2414314624007429

**Published:** 2024-08-09

**Authors:** Xueqing Song, Woldegebriel Yeibyo, William Li, Robert D. Pike

**Affiliations:** a University of the District of Columbia, Division of Sciences and Mathematics 4200 Connecticut Avenue NW 20008 Washington DC USA; b College of William & Mary, Department of Chemistry, 540 Landrum Drive, Williamsburg, VA 23185, USA; Sunway University, Malaysia

**Keywords:** crystal structure, hydrogen bonding, salt, *cis*-trigonal–bipyramid coordination

## Abstract

Crystals of the title salt, (C_8_H_20_N)[Sn(C_6_H_5_)_3_(C_2_H_2_O_2_S)], comprise diisobutyl­ammonium cations and mercapto­acetato­tri­phenyl­stannate(IV) anions. The bidentate binding mode of the mercapto­acetate ligand gives rise to a five-coordinated, ionic tri­phenyl­tin complex with a distorted *cis*-trigonal–bipyramidal geometry around the tin atom. In the crystal, charge-assisted ammonium-N—H⋯O(carboxyl­ate) hydrogen-bonding connects two cations and two anions into a four-ion aggregate. Two positions were resolved for one of the phenyl rings with the major component having a site occupancy factor of 0.60 (3).

## Abstract 2

The title salt features a distorted *cis*-trigonal-bipyramid coordination geometry around the tin atom.

## Graphical abstract

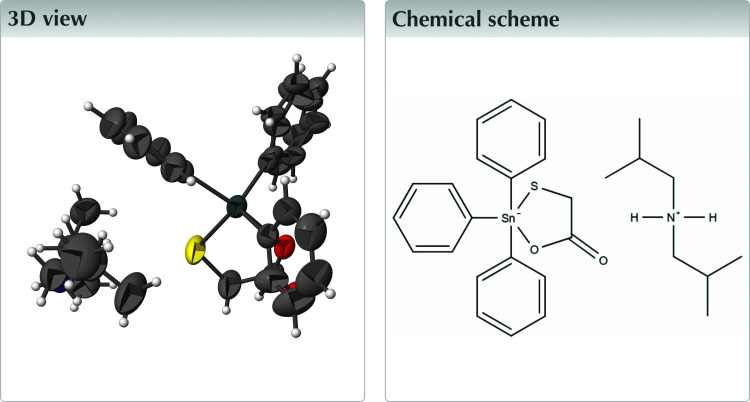

## Structure description

**Figure 1:**
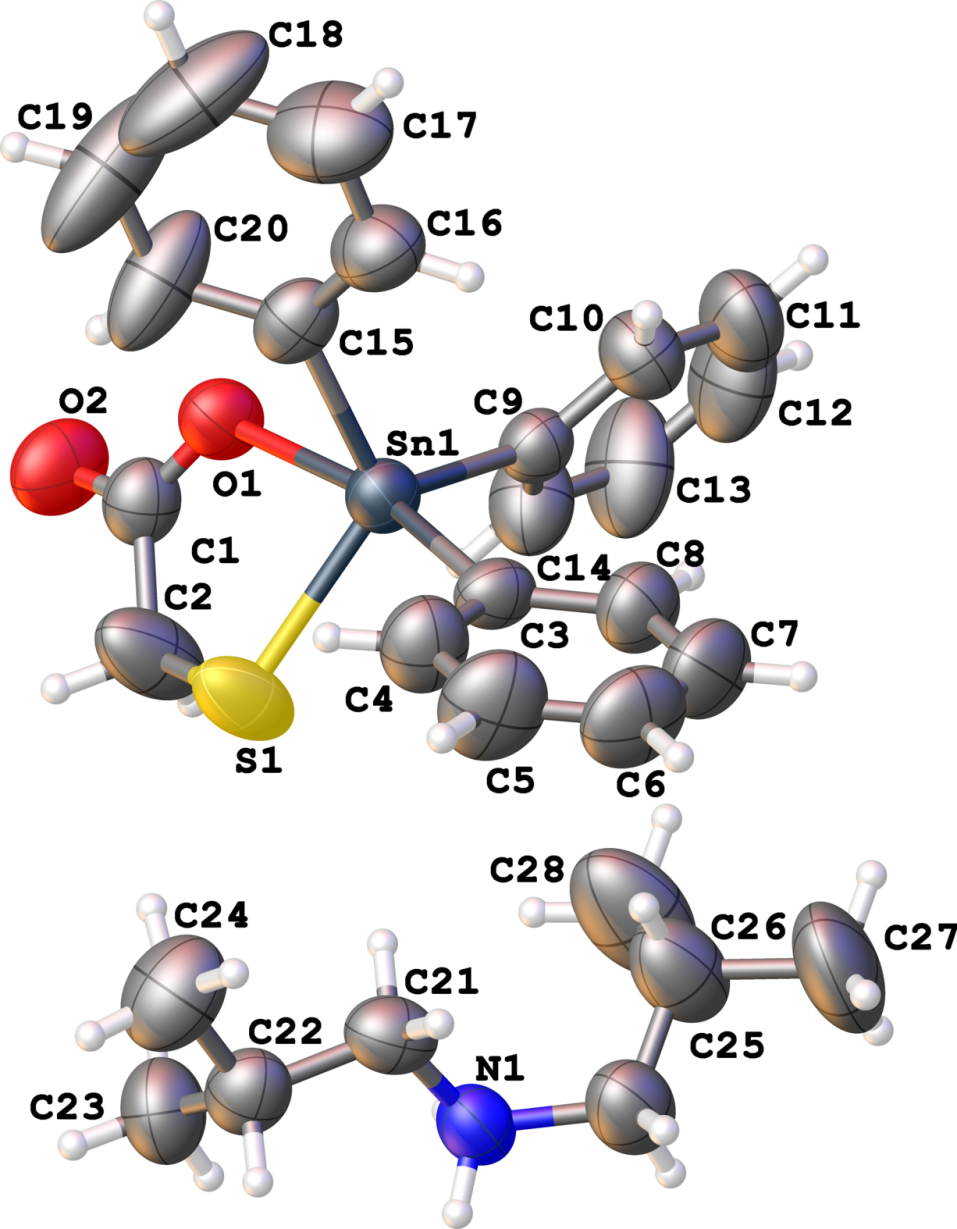
The mol­ecular structures of the two ions comprising the asymmetric unit in the title salt showing the atom-labelling scheme and anisotropic displacement ellipsoids at the 50% probability level.

One of the authors has reported some ionic di- and triorganotin complexes (Song *et al.*, 2005 ▸, 2005 ▸; Zhong *et al.*, 2005 ▸) as part of efforts to modify structures of organotin complexes with the aim to increase their aqueous solubility (Gielen, 2002 ▸). The title salt is another example of an ionic tri­phenyl­tin complex of mercapto­acetic acid. The salt comprises a diisobutyl­ammonium cation and a mercapto­acetato­tri­phenyl­stannate anion, Fig. 1 ▸. This new species is similar to other reported ionic complexes like di-*cyclo*-hexyl­ammonium thiol­actatotri­phenyl­stannate (Song *et al.*, 2006 ▸), tri­methyl­ammonium chlorido­diphenyl­mercapto­acetato­stannnate (Song *et al.*, 2005 ▸) and 2-methyl­pyrimidium chlorido­diphenyl­mercaptostannate (Zhong *et al.*, 2005 ▸). In the new salt, the Ph_3_Sn residue is covalently bound to a sulfur atom (Sn1—S1 2.423 (1) Å) and also to a less tightly bound carboxyl­ate-O atom as indicated by the relatively long Sn1—O1 bond length of 2.456 (2) Å. The coordination environment around the Sn atom is based on a distorted *cis*-trigonal–bipyramidal geometry with the O1 and C3 atoms in the axial positions, and the S1, C9 and C15 atoms in the equatorial plane. The axial axis is bent with the C3—Sn1—O1 angle being 168.74 (10)°. The S1—Sn1—O1 angle is significantly reduced [75.58 (6)°] from 90° due to the restricted bite distance of the mercapto­acetato ligand.

**Figure 2:**
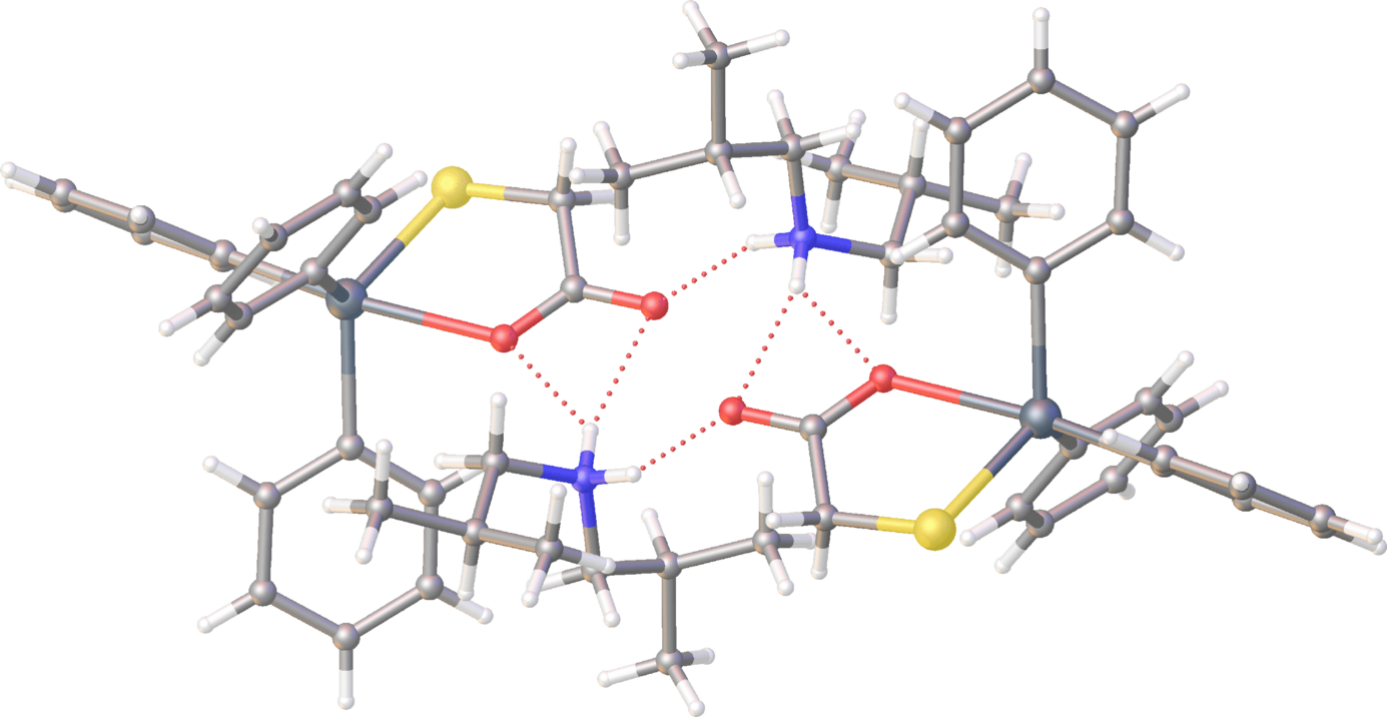
A view of the four-ion aggregate in the crystal of the title salt. Dashed lines indicate hydrogen bonds.

Charge-assisted hydrogen-bonding inter­actions (Table 1 ▸) between the di-*iso-*butyl­ammonium cations and the mercapto­acetato­tri­phenyl­stannate anions are observed in the crystal. As shown in Fig. 2 ▸, one ammonium-N—H atom forms a hydrogen bond to the carbonyl-O atom of one carboxyl­ate residue and the second ammonium-N—H atom bifurcates the carboxyl-O atom of a centrosymmetrically related carboxyl­ate residue. In this way, a four-ion aggregate is formed with a central twelve-membered {⋯HNH⋯OCO}_2_ synthon with the outer N—H⋯O hydrogen bonds lying above and below the encompassed eight-membered {⋯HNH⋯O}_2_ synthon. The hydrogen bonding substanti­ally affects the distribution of the electrons within the carboxyl­ate group, which can be seen by the observation of experimentally equivalent C—O bond lengths, *i.e*. 1.238 (4) Å for the C1—O1 bond and 1.241 (4) Å for the C1—O2 bond.

## Synthesis and crystallization

The salt was prepared by the addition, with stirring, of mercapto­acetic acid (2 mmol) to an acetone solution (30 ml) containing tri­phenyl­tin hydroxide (2 mmol). To this solution was added, drop-wise, di-*iso-*butyl­amine (2 mmol) in acetone (20 ml). A cloudy solution formed immediately and after refluxing for 2 h, the solution became clear. The crude product was obtained as a solid by removing the solvent on a rotary evaporator. It was then recrystallized from 95% ethanol and upon slow evaporation crystals suitable for X-ray diffraction analysis were obtained. Yield 71%, m.p. 108–109 °C.

## Refinement

Crystal data, data collection and structure refinement details are summarized in Table 2 ▸. Two positions, in a ratio 0.60 (3):0.40 (3), were resolved for the C15—C20 phenyl ring; all C atoms were refined with anisotropic displacement parameters.

## Supplementary Material

Crystal structure: contains datablock(s) I. DOI: 10.1107/S2414314624007429/tk4107sup1.cif

Structure factors: contains datablock(s) I. DOI: 10.1107/S2414314624007429/tk4107Isup2.hkl

Supporting information file. DOI: 10.1107/S2414314624007429/tk4107Isup3.mol

CCDC reference: 299548

Additional supporting information:  crystallographic information; 3D view; checkCIF report

## Figures and Tables

**Table 1:** Hydrogen-bond geometry (Å, °)

*D*—H⋯*A*	*D*—H	H⋯*A*	*D*⋯*A*	*D*—H⋯*A*
N1—H1*A*⋯O2^i^	0.89	1.95	2.791 (4)	157
N1—H1*B*⋯O1^ii^	0.89	2.16	2.894 (3)	140
N1—H1*B*⋯O2^ii^	0.89	2.23	3.072 (4)	158

Symmetry codes: (i) 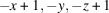; (ii) 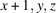.

**Table 2:** Experimental details

Crystal data	
Chemical formula	(C_8_H_20_N)[Sn(C_6_H_5_)_3_(C_2_H_2_O_2_S)]
*M*_r_	570.33
Crystal system, space group	Monoclinic, *P*2_1_/*n*
Temperature (K)	296
*a*, *b*, *c* (Å)	10.4032 (1), 18.8988 (3), 14.8277 (2)
β (°)	101.382 (1)
*V* (Å^3^)	2857.91 (7)
*Z*	4
Radiation type	Cu *K*α
μ (mm^−1^)	7.96
Crystal size (mm)	0.39 × 0.25 × 0.18

Data collection	
Diffractometer	Bruker APEXII CCD
Absorption correction	Multi-scan (*SADABS*; Krause *et al.*, 2015 ▸)
*T*_min_, *T*_max_	0.439, 0.753
No. of measured, independent and observed [*I* > 2σ(*I*)] reflections	35046, 5313, 4767
*R*_int_	0.040
(sin θ/λ)_max_ (Å^−1^)	0.612

Refinement	
*R*[*F*^2^ > 2σ(*F*^2^)], *wR*(*F*^2^), *S*	0.032, 0.082, 1.09
No. of reflections	5313
No. of parameters	358
No. of restraints	287
H-atom treatment	H-atom parameters constrained
Δρ_max_, Δρ_min_ (e Å^−3^)	0.49, −0.45

Computer programs: *APEX2* and *SAINT-Plus* (Bruker, 2004 ▸), *SHELXS97* (Sheldrick, 2008 ▸), *SHELXL2019/3* (Sheldrick, 2015 ▸) and *OLEX2* (Dolomanov *et al.*, 2009 ▸).

## full crystallographic data

### Diisobutylammonium triphenyl(2-thiolatoacetato-κ2O,S)stannate(IV) Crystal data

**Table:**

(C_8_H_20_N)[Sn(C_6_H_5_)_3_(C_2_H_2_O_2_S)]	*F*(000) = 1176
*M**_r_* = 570.33	*D*_x_ = 1.326 Mg m^−^^3^
Monoclinic, *P*2_1_/*n*	Cu *K*α radiation, λ = 1.54178 Å
*a* = 10.4032 (1) Å	Cell parameters from 9852 reflections
*b* = 18.8988 (3) Å	θ = 3.0–70.1°
*c* = 14.8277 (2) Å	µ = 7.96 mm^−^^1^
β = 101.382 (1)°	*T* = 296 K
*V* = 2857.91 (7) Å^3^	Block, colourless
*Z* = 4	0.39 × 0.25 × 0.18 mm

### Diisobutylammonium triphenyl(2-thiolatoacetato-κ2O,S)stannate(IV) Data collection

**Table:**

Bruker APEXII CCD diffractometer	4767 reflections with *I* > 2σ(*I*)
ω and Phi scans	*R*_int_ = 0.040
Absorption correction: multi-scan (SADABS; Krause *et al.*, 2015)	θ_max_ = 70.6°, θ_min_ = 3.8°
*T*_min_ = 0.439, *T*_max_ = 0.753	*h* = −11→12
35046 measured reflections	*k* = −22→22
5313 independent reflections	*l* = −16→17

### Diisobutylammonium triphenyl(2-thiolatoacetato-κ2O,S)stannate(IV) Refinement

**Table:**

Refinement on *F*^2^	Hydrogen site location: inferred from neighbouring sites
Least-squares matrix: full	H-atom parameters constrained
*R*[*F*^2^ > 2σ(*F*^2^)] = 0.032	*w* = 1/[σ^2^(*F*_o_^2^) + (0.0367*P*)^2^ + 1.6022*P*] where *P* = (*F*_o_^2^ + 2*F*_c_^2^)/3
*wR*(*F*^2^) = 0.082	(Δ/σ)_max_ = 0.001
*S* = 1.09	Δρ_max_ = 0.49 e Å^−^^3^
5313 reflections	Δρ_min_ = −0.45 e Å^−^^3^
358 parameters	Extinction correction: *SHELXL2019/3* (Sheldrick, 2015), Fc^*^=kFc[1+0.001xFc^2^λ^3^/sin(2θ)]^-1/4^
287 restraints	Extinction coefficient: 0.00187 (8)

### Diisobutylammonium triphenyl(2-thiolatoacetato-κ2O,S)stannate(IV) Special details

**Table:**

Geometry. All esds (except the esd in the dihedral angle between two l.s. planes) are estimated using the full covariance matrix. The cell esds are taken into account individually in the estimation of esds in distances, angles and torsion angles; correlations between esds in cell parameters are only used when they are defined by crystal symmetry. An approximate (isotropic) treatment of cell esds is used for estimating esds involving l.s. planes.

### Diisobutylammonium triphenyl(2-thiolatoacetato-κ2O,S)stannate(IV) Fractional atomic coordinates and isotropic or equivalent isotropic displacement parameters (Å^2^)

**Table:**

	*x*	*y*	*z*	*U*_iso_*/*U*_eq_	Occ. (<1)
Sn1	0.29678 (2)	0.09786 (2)	0.24494 (2)	0.05419 (10)	
S1	0.45120 (9)	0.10232 (6)	0.39021 (6)	0.0876 (3)	
O1	0.1637 (2)	0.07224 (13)	0.36093 (16)	0.0666 (5)	
O2	0.1506 (3)	0.05058 (15)	0.50356 (18)	0.0860 (7)	
C1	0.2142 (3)	0.06407 (17)	0.4431 (2)	0.0631 (7)	
C2	0.3620 (4)	0.0666 (3)	0.4722 (3)	0.1027 (15)	
H2A	0.393128	0.018846	0.487275	0.123*	
H2B	0.383473	0.094437	0.528113	0.123*	
C3	0.4402 (3)	0.13121 (18)	0.1641 (2)	0.0588 (7)	
C4	0.5037 (4)	0.1952 (2)	0.1820 (3)	0.0813 (10)	
H4	0.485694	0.223468	0.229340	0.098*	
C5	0.5941 (5)	0.2185 (3)	0.1309 (4)	0.1038 (14)	
H5	0.635244	0.261967	0.144120	0.125*	
C6	0.6225 (4)	0.1781 (3)	0.0618 (3)	0.0991 (13)	
H6	0.683038	0.193809	0.027787	0.119*	
C7	0.5623 (4)	0.1147 (3)	0.0424 (3)	0.0944 (12)	
H7	0.581509	0.086972	−0.004996	0.113*	
C8	0.4714 (4)	0.0910 (2)	0.0936 (3)	0.0771 (10)	
H8	0.431091	0.047333	0.079869	0.093*	
C9	0.2351 (3)	−0.00545 (16)	0.1959 (2)	0.0563 (7)	
C10	0.1733 (4)	−0.0159 (2)	0.1058 (2)	0.0755 (9)	
H10	0.159273	0.022345	0.065598	0.091*	
C14	0.2544 (5)	−0.0634 (2)	0.2534 (3)	0.0848 (10)	
H14	0.296426	−0.057856	0.314441	0.102*	
C11	0.1315 (5)	−0.0834 (2)	0.0741 (3)	0.0971 (13)	
H11	0.090190	−0.090171	0.013162	0.117*	
C12	0.1517 (5)	−0.1389 (2)	0.1332 (4)	0.1092 (15)	
H12	0.123532	−0.183854	0.112612	0.131*	
C13	0.2123 (6)	−0.1294 (2)	0.2215 (4)	0.1142 (17)	
H13	0.225776	−0.167915	0.261307	0.137*	
C15A	0.160 (2)	0.1774 (13)	0.2172 (12)	0.057 (3)	0.60 (3)
C16A	0.1154 (19)	0.1948 (9)	0.1277 (12)	0.071 (4)	0.60 (3)
H16A	0.144453	0.168986	0.082169	0.086*	0.60 (3)
C17A	0.0257 (18)	0.2514 (9)	0.1015 (11)	0.109 (5)	0.60 (3)
H17A	0.000005	0.263945	0.039920	0.131*	0.60 (3)
C18A	−0.0224 (17)	0.2872 (8)	0.1682 (13)	0.104 (4)	0.60 (3)
H18A	−0.084414	0.322806	0.152316	0.125*	0.60 (3)
C19A	0.0231 (17)	0.2694 (5)	0.2595 (11)	0.092 (4)	0.60 (3)
H19A	−0.008020	0.293885	0.305199	0.110*	0.60 (3)
C20A	0.1138 (17)	0.2160 (6)	0.2838 (10)	0.074 (3)	0.60 (3)
H20A	0.144433	0.205754	0.345658	0.089*	0.60 (3)
C15B	0.140 (3)	0.1803 (16)	0.197 (2)	0.050 (4)	0.40 (3)
C16B	0.121 (3)	0.2043 (15)	0.1048 (16)	0.069 (4)	0.40 (3)
H16B	0.176950	0.188357	0.067684	0.083*	0.40 (3)
C17B	0.024 (2)	0.2491 (11)	0.0687 (17)	0.087 (5)	0.40 (3)
H17B	0.011387	0.260865	0.006694	0.104*	0.40 (3)
C18B	−0.0521 (18)	0.2767 (12)	0.120 (2)	0.102 (6)	0.40 (3)
H18B	−0.119488	0.307409	0.095081	0.123*	0.40 (3)
C19B	−0.032 (3)	0.2601 (14)	0.210 (2)	0.117 (9)	0.40 (3)
H19B	−0.083472	0.281884	0.247046	0.141*	0.40 (3)
C20B	0.064 (2)	0.2111 (12)	0.2513 (18)	0.083 (6)	0.40 (3)
H20B	0.075348	0.199972	0.313465	0.100*	0.40 (3)
N1	0.8901 (3)	0.04696 (15)	0.36359 (18)	0.0650 (6)	
H1A	0.853689	0.019449	0.400328	0.078*	
H1B	0.972417	0.054949	0.392118	0.078*	
C21	0.8199 (3)	0.11575 (19)	0.3547 (2)	0.0697 (8)	
H21A	0.726345	0.106843	0.345475	0.084*	
H21B	0.836613	0.140108	0.300649	0.084*	
C22	0.8592 (4)	0.16325 (19)	0.4370 (3)	0.0730 (9)	
H22	0.953152	0.173206	0.444635	0.088*	
C23	0.8347 (5)	0.1307 (2)	0.5254 (3)	0.0935 (12)	
H23A	0.743133	0.119985	0.518959	0.140*	
H23B	0.860454	0.163490	0.575101	0.140*	
H23C	0.884953	0.087993	0.538063	0.140*	
C24	0.7845 (5)	0.2333 (2)	0.4176 (4)	0.1104 (15)	
H24A	0.794891	0.251289	0.358927	0.166*	
H24B	0.818729	0.266898	0.464762	0.166*	
H24C	0.693144	0.225458	0.416926	0.166*	
C25	0.8944 (4)	0.0058 (2)	0.2778 (3)	0.0893 (11)	
H25A	0.941560	0.033477	0.239737	0.107*	
H25B	0.943907	−0.037271	0.294836	0.107*	
C26	0.7681 (5)	−0.0129 (3)	0.2235 (3)	0.1031 (14)	
H26	0.723996	0.031675	0.202671	0.124*	
C27	0.7886 (6)	−0.0520 (4)	0.1363 (4)	0.150 (2)	
H27A	0.838026	−0.022483	0.102841	0.225*	
H27B	0.704961	−0.062563	0.098270	0.225*	
H27C	0.835626	−0.095166	0.153432	0.225*	
C28	0.6779 (6)	−0.0524 (4)	0.2708 (4)	0.148 (3)	
H28A	0.714823	−0.097878	0.289587	0.222*	
H28B	0.595003	−0.058588	0.229658	0.222*	
H28C	0.665230	−0.026345	0.323974	0.222*	

### Diisobutylammonium triphenyl(2-thiolatoacetato-κ2O,S)stannate(IV) Atomic displacement parameters (Å^2^)

**Table:**

	*U*^11^	*U*^22^	*U*^33^	*U*^12^	*U*^13^	*U*^23^
Sn1	0.05088 (14)	0.06135 (14)	0.05259 (14)	−0.00563 (8)	0.01566 (8)	−0.00136 (8)
S1	0.0602 (5)	0.1427 (9)	0.0589 (5)	−0.0359 (5)	0.0091 (4)	0.0025 (5)
O1	0.0582 (12)	0.0821 (14)	0.0624 (13)	−0.0099 (11)	0.0194 (10)	0.0025 (11)
O2	0.0839 (17)	0.106 (2)	0.0791 (16)	0.0043 (14)	0.0415 (13)	0.0217 (14)
C1	0.0700 (19)	0.0609 (18)	0.0635 (19)	−0.0077 (15)	0.0258 (15)	−0.0004 (14)
C2	0.078 (2)	0.172 (4)	0.057 (2)	−0.034 (3)	0.0115 (18)	0.014 (2)
C3	0.0458 (15)	0.0736 (19)	0.0594 (17)	0.0009 (13)	0.0162 (13)	0.0063 (14)
C4	0.081 (2)	0.083 (2)	0.087 (3)	−0.0152 (19)	0.036 (2)	−0.0066 (19)
C5	0.094 (3)	0.100 (3)	0.129 (4)	−0.030 (2)	0.051 (3)	0.000 (3)
C6	0.082 (3)	0.126 (4)	0.103 (3)	−0.006 (2)	0.052 (2)	0.018 (3)
C7	0.086 (3)	0.126 (3)	0.083 (3)	0.002 (2)	0.045 (2)	−0.005 (2)
C8	0.074 (2)	0.090 (3)	0.073 (2)	−0.0051 (18)	0.0302 (18)	−0.0067 (17)
C9	0.0525 (16)	0.0599 (16)	0.0597 (16)	0.0008 (13)	0.0190 (13)	−0.0039 (13)
C10	0.080 (2)	0.077 (2)	0.068 (2)	−0.0079 (18)	0.0109 (17)	−0.0059 (17)
C14	0.120 (3)	0.064 (2)	0.073 (2)	0.012 (2)	0.026 (2)	0.0054 (16)
C11	0.100 (3)	0.098 (3)	0.093 (3)	−0.019 (2)	0.017 (2)	−0.034 (2)
C12	0.142 (4)	0.067 (2)	0.132 (4)	−0.019 (3)	0.060 (3)	−0.029 (2)
C13	0.188 (5)	0.055 (2)	0.113 (3)	0.004 (3)	0.063 (3)	0.000 (2)
C15A	0.052 (6)	0.059 (6)	0.061 (7)	−0.001 (4)	0.018 (6)	0.004 (5)
C16A	0.083 (5)	0.061 (7)	0.071 (8)	0.015 (5)	0.020 (6)	0.009 (5)
C17A	0.132 (9)	0.105 (7)	0.087 (8)	0.053 (7)	0.011 (7)	0.013 (6)
C18A	0.117 (9)	0.087 (7)	0.117 (9)	0.052 (7)	0.047 (7)	0.033 (6)
C19A	0.120 (9)	0.069 (5)	0.103 (7)	0.031 (5)	0.064 (7)	0.019 (5)
C20A	0.096 (9)	0.053 (4)	0.082 (6)	0.013 (5)	0.040 (5)	0.009 (4)
C15B	0.052 (9)	0.041 (5)	0.060 (10)	−0.001 (5)	0.014 (8)	0.019 (7)
C16B	0.076 (7)	0.072 (7)	0.057 (8)	−0.010 (5)	0.008 (6)	0.028 (6)
C17B	0.081 (7)	0.086 (8)	0.085 (11)	−0.011 (6)	−0.002 (6)	0.031 (7)
C18B	0.073 (8)	0.100 (11)	0.137 (14)	0.005 (7)	0.027 (8)	0.058 (11)
C19B	0.106 (13)	0.121 (14)	0.144 (16)	0.059 (11)	0.072 (12)	0.068 (13)
C20B	0.075 (10)	0.091 (10)	0.096 (11)	0.022 (8)	0.048 (8)	0.043 (9)
N1	0.0616 (15)	0.0770 (17)	0.0565 (15)	−0.0099 (13)	0.0118 (12)	−0.0065 (12)
C21	0.0609 (19)	0.082 (2)	0.0644 (19)	−0.0054 (16)	0.0091 (15)	0.0126 (16)
C22	0.067 (2)	0.071 (2)	0.084 (2)	−0.0069 (16)	0.0229 (17)	−0.0045 (17)
C23	0.112 (3)	0.098 (3)	0.075 (2)	−0.003 (2)	0.029 (2)	−0.009 (2)
C24	0.124 (4)	0.082 (3)	0.136 (4)	0.011 (3)	0.051 (3)	0.008 (3)
C25	0.084 (3)	0.112 (3)	0.075 (2)	−0.015 (2)	0.0233 (19)	−0.012 (2)
C26	0.096 (3)	0.144 (4)	0.072 (2)	−0.034 (3)	0.021 (2)	−0.009 (2)
C27	0.162 (5)	0.208 (7)	0.081 (3)	−0.039 (5)	0.025 (3)	−0.055 (4)
C28	0.135 (5)	0.196 (7)	0.113 (4)	−0.088 (5)	0.025 (3)	−0.015 (4)

### Diisobutylammonium triphenyl(2-thiolatoacetato-κ2O,S)stannate(IV) Geometric parameters (Å, º)

**Table:**

Sn1—S1	2.4212 (9)	C19A—H19A	0.9300
Sn1—O1	2.459 (2)	C19A—C20A	1.381 (12)
Sn1—C3	2.183 (3)	C20A—H20A	0.9300
Sn1—C9	2.137 (3)	C15B—C16B	1.411 (19)
Sn1—C15A	2.055 (19)	C15B—C20B	1.371 (18)
Sn1—C15B	2.27 (2)	C16B—H16B	0.9300
S1—C2	1.800 (4)	C16B—C17B	1.34 (3)
O1—C1	1.238 (4)	C17B—H17B	0.9300
O2—C1	1.241 (4)	C17B—C18B	1.32 (3)
C1—C2	1.513 (5)	C18B—H18B	0.9300
C2—H2A	0.9700	C18B—C19B	1.35 (2)
C2—H2B	0.9700	C19B—H19B	0.9300
C3—C4	1.379 (5)	C19B—C20B	1.409 (19)
C3—C8	1.382 (5)	C20B—H20B	0.9300
C4—H4	0.9300	N1—H1A	0.8900
C4—C5	1.390 (5)	N1—H1B	0.8900
C5—H5	0.9300	N1—C21	1.484 (4)
C5—C6	1.356 (6)	N1—C25	1.499 (4)
C6—H6	0.9300	C21—H21A	0.9700
C6—C7	1.355 (6)	C21—H21B	0.9700
C7—H7	0.9300	C21—C22	1.505 (5)
C7—C8	1.397 (5)	C22—H22	0.9800
C8—H8	0.9300	C22—C23	1.513 (5)
C9—C10	1.378 (5)	C22—C24	1.533 (6)
C9—C14	1.379 (5)	C23—H23A	0.9600
C10—H10	0.9300	C23—H23B	0.9600
C10—C11	1.399 (5)	C23—H23C	0.9600
C14—H14	0.9300	C24—H24A	0.9600
C14—C13	1.374 (6)	C24—H24B	0.9600
C11—H11	0.9300	C24—H24C	0.9600
C11—C12	1.355 (7)	C25—H25A	0.9700
C12—H12	0.9300	C25—H25B	0.9700
C12—C13	1.350 (7)	C25—C26	1.444 (6)
C13—H13	0.9300	C26—H26	0.9800
C15A—C16A	1.356 (14)	C26—C27	1.539 (6)
C15A—C20A	1.388 (15)	C26—C28	1.480 (6)
C16A—H16A	0.9300	C27—H27A	0.9600
C16A—C17A	1.422 (19)	C27—H27B	0.9600
C17A—H17A	0.9300	C27—H27C	0.9600
C17A—C18A	1.372 (15)	C28—H28A	0.9600
C18A—H18A	0.9300	C28—H28B	0.9600
C18A—C19A	1.385 (13)	C28—H28C	0.9600
			
S1—Sn1—O1	75.58 (6)	C15A—C20A—H20A	119.6
C3—Sn1—S1	94.18 (8)	C19A—C20A—C15A	120.9 (10)
C3—Sn1—O1	168.74 (10)	C19A—C20A—H20A	119.6
C3—Sn1—C15B	98.9 (7)	C16B—C15B—Sn1	119.0 (17)
C9—Sn1—S1	115.87 (9)	C20B—C15B—Sn1	124.6 (15)
C9—Sn1—O1	83.40 (9)	C20B—C15B—C16B	116.4 (18)
C9—Sn1—C3	105.62 (12)	C15B—C16B—H16B	118.6
C9—Sn1—C15B	111.8 (10)	C17B—C16B—C15B	123 (2)
C15A—Sn1—S1	117.7 (6)	C17B—C16B—H16B	118.6
C15A—Sn1—O1	79.7 (5)	C16B—C17B—H17B	119.7
C15A—Sn1—C3	101.6 (6)	C18B—C17B—C16B	120.5 (18)
C15A—Sn1—C9	116.7 (8)	C18B—C17B—H17B	119.7
C15B—Sn1—S1	124.5 (9)	C17B—C18B—H18B	120.3
C15B—Sn1—O1	83.6 (7)	C17B—C18B—C19B	119.4 (16)
C2—S1—Sn1	104.34 (13)	C19B—C18B—H18B	120.3
C1—O1—Sn1	121.7 (2)	C18B—C19B—H19B	118.7
O1—C1—O2	123.6 (3)	C18B—C19B—C20B	122.5 (16)
O1—C1—C2	119.1 (3)	C20B—C19B—H19B	118.7
O2—C1—C2	117.2 (3)	C15B—C20B—C19B	117.9 (16)
S1—C2—H2A	108.1	C15B—C20B—H20B	121.0
S1—C2—H2B	108.1	C19B—C20B—H20B	121.0
C1—C2—S1	116.7 (3)	H1A—N1—H1B	107.1
C1—C2—H2A	108.1	C21—N1—H1A	107.7
C1—C2—H2B	108.1	C21—N1—H1B	107.7
H2A—C2—H2B	107.3	C21—N1—C25	118.4 (3)
C4—C3—Sn1	120.1 (2)	C25—N1—H1A	107.7
C4—C3—C8	116.9 (3)	C25—N1—H1B	107.7
C8—C3—Sn1	123.0 (3)	N1—C21—H21A	108.8
C3—C4—H4	119.3	N1—C21—H21B	108.8
C3—C4—C5	121.5 (4)	N1—C21—C22	113.6 (3)
C5—C4—H4	119.3	H21A—C21—H21B	107.7
C4—C5—H5	119.8	C22—C21—H21A	108.8
C6—C5—C4	120.4 (4)	C22—C21—H21B	108.8
C6—C5—H5	119.8	C21—C22—H22	108.2
C5—C6—H6	120.1	C21—C22—C23	113.2 (3)
C7—C6—C5	119.8 (4)	C21—C22—C24	108.4 (3)
C7—C6—H6	120.1	C23—C22—H22	108.2
C6—C7—H7	119.9	C23—C22—C24	110.4 (3)
C6—C7—C8	120.1 (4)	C24—C22—H22	108.2
C8—C7—H7	119.9	C22—C23—H23A	109.5
C3—C8—C7	121.3 (4)	C22—C23—H23B	109.5
C3—C8—H8	119.4	C22—C23—H23C	109.5
C7—C8—H8	119.4	H23A—C23—H23B	109.5
C10—C9—Sn1	120.8 (2)	H23A—C23—H23C	109.5
C10—C9—C14	118.0 (3)	H23B—C23—H23C	109.5
C14—C9—Sn1	121.2 (3)	C22—C24—H24A	109.5
C9—C10—H10	119.6	C22—C24—H24B	109.5
C9—C10—C11	120.7 (4)	C22—C24—H24C	109.5
C11—C10—H10	119.6	H24A—C24—H24B	109.5
C9—C14—H14	119.6	H24A—C24—H24C	109.5
C13—C14—C9	120.7 (4)	H24B—C24—H24C	109.5
C13—C14—H14	119.6	N1—C25—H25A	108.5
C10—C11—H11	120.3	N1—C25—H25B	108.5
C12—C11—C10	119.4 (4)	H25A—C25—H25B	107.5
C12—C11—H11	120.3	C26—C25—N1	115.1 (3)
C11—C12—H12	119.8	C26—C25—H25A	108.5
C13—C12—C11	120.5 (4)	C26—C25—H25B	108.5
C13—C12—H12	119.8	C25—C26—H26	106.4
C14—C13—H13	119.6	C25—C26—C27	108.9 (4)
C12—C13—C14	120.7 (4)	C25—C26—C28	116.9 (4)
C12—C13—H13	119.6	C27—C26—H26	106.4
C16A—C15A—Sn1	117.6 (11)	C28—C26—H26	106.4
C16A—C15A—C20A	118.0 (12)	C28—C26—C27	111.3 (5)
C20A—C15A—Sn1	124.4 (10)	C26—C27—H27A	109.5
C15A—C16A—H16A	119.0	C26—C27—H27B	109.5
C15A—C16A—C17A	121.9 (12)	C26—C27—H27C	109.5
C17A—C16A—H16A	119.0	H27A—C27—H27B	109.5
C16A—C17A—H17A	120.4	H27A—C27—H27C	109.5
C18A—C17A—C16A	119.2 (11)	H27B—C27—H27C	109.5
C18A—C17A—H17A	120.4	C26—C28—H28A	109.5
C17A—C18A—H18A	120.6	C26—C28—H28B	109.5
C17A—C18A—C19A	118.8 (10)	C26—C28—H28C	109.5
C19A—C18A—H18A	120.6	H28A—C28—H28B	109.5
C18A—C19A—H19A	119.4	H28A—C28—H28C	109.5
C20A—C19A—C18A	121.1 (8)	H28B—C28—H28C	109.5
C20A—C19A—H19A	119.4		
			
Sn1—S1—C2—C1	−17.7 (4)	C10—C11—C12—C13	−0.4 (8)
Sn1—O1—C1—O2	−179.5 (3)	C14—C9—C10—C11	0.2 (6)
Sn1—O1—C1—C2	−2.8 (5)	C11—C12—C13—C14	0.2 (8)
Sn1—C3—C4—C5	−179.0 (3)	C15A—C16A—C17A—C18A	−3 (3)
Sn1—C3—C8—C7	179.0 (3)	C16A—C15A—C20A—C19A	1 (3)
Sn1—C9—C10—C11	−179.2 (3)	C16A—C17A—C18A—C19A	3 (3)
Sn1—C9—C14—C13	178.9 (3)	C17A—C18A—C19A—C20A	−1 (2)
Sn1—C15A—C16A—C17A	−176.8 (17)	C18A—C19A—C20A—C15A	−2 (2)
Sn1—C15A—C20A—C19A	179.0 (13)	C20A—C15A—C16A—C17A	1 (3)
Sn1—C15B—C16B—C17B	175 (2)	C15B—C16B—C17B—C18B	5 (4)
Sn1—C15B—C20B—C19B	−178.2 (19)	C16B—C15B—C20B—C19B	4 (4)
O1—C1—C2—S1	14.6 (6)	C16B—C17B—C18B—C19B	1 (3)
O2—C1—C2—S1	−168.6 (3)	C17B—C18B—C19B—C20B	−3 (3)
C3—C4—C5—C6	−0.3 (7)	C18B—C19B—C20B—C15B	1 (3)
C4—C3—C8—C7	−0.6 (6)	C20B—C15B—C16B—C17B	−7 (4)
C4—C5—C6—C7	0.1 (8)	N1—C21—C22—C23	−59.6 (4)
C5—C6—C7—C8	−0.1 (7)	N1—C21—C22—C24	177.6 (3)
C6—C7—C8—C3	0.3 (7)	N1—C25—C26—C27	177.7 (4)
C8—C3—C4—C5	0.5 (6)	N1—C25—C26—C28	−55.2 (7)
C9—C10—C11—C12	0.3 (7)	C21—N1—C25—C26	−58.7 (5)
C9—C14—C13—C12	0.3 (7)	C25—N1—C21—C22	−160.1 (3)
C10—C9—C14—C13	−0.5 (6)		

### Diisobutylammonium triphenyl(2-thiolatoacetato-κ2O,S)stannate(IV) Hydrogen-bond geometry (Å, º)

**Table:**

*D*—H···*A*	*D*—H	H···*A*	*D*···*A*	*D*—H···*A*
N1—H1*A*···O2^i^	0.89	1.95	2.791 (4)	157
N1—H1*B*···O1^ii^	0.89	2.16	2.894 (3)	140
N1—H1*B*···O2^ii^	0.89	2.23	3.072 (4)	158

Symmetry codes: (i) −*x*+1, −*y*, −*z*+1; (ii) *x*+1, *y*, *z*.
